# Advancing Transbronchial Lung Cryobiopsy in Interstitial Lung Disease with Adjunctive Tools and Smaller Cryoprobes

**DOI:** 10.3390/jcm15135061

**Published:** 2026-06-29

**Authors:** Rosa Arancibia-Cacace, Sultana Alam, Michelle Siew

**Affiliations:** Division of Pulmonary and Critical Care Medicine, SUNY Downstate Health Sciences University, 450 Clarkson Avenue, Brooklyn, NY 11203, USA

**Keywords:** interstitial lung disease, transbronchial lung cryobiopsy, cryoprobes, 1.1 mm and 1.7 mm cryoprobes

## Abstract

Transbronchial lung cryobiopsy (TBLC) is increasingly used as a minimally invasive approach for tissue acquisition in the evaluation of interstitial lung disease (ILD), serving as an alternative to surgical lung biopsy (SLB) within multidisciplinary diagnostic pathways. Despite its growing adoption, variability in diagnostic yield and complication rates highlight the importance of procedural technique, probe selection, and freezing parameters. This narrative review summarizes the current landscape of TBLC, with emphasis on factors that influence diagnostic performance and safety, including procedural considerations involving endobronchial balloon blockade (EBB), radial probe endobronchial ultrasound (RP-EBUS), and cone-beam computed tomography (CBCT) for biopsy localization and airway management. Much of the existing experience is based on conventional cryoprobes, including 2.4 mm and 1.9 mm devices, typically used with freezing times of several seconds. While these approaches have defined the current role of TBLC in ILD, outcomes remain variable across centers, prompting continued refinement of procedural strategies to improve consistency. More recently, attention has expanded to include a broader range of smaller cryoprobe sizes—1.7 mm and 1.1 mm. Overall, this review provides a framework for understanding contemporary TBLC practice and highlights key areas where further study is needed to better define optimal technique and improve consistency in clinical outcomes.

## 1. Introduction

Interstitial lung diseases (ILDs) are a group of diseases characterized by inflammation and scarring of the lung parenchyma [1,2] but differ in etiology, pathological findings, treatment options and prognosis [1,3]. In patients with suspected ILD, the clinical and imaging findings of chest radiography and conventional computed tomography are generally nonspecific and performing high-resolution computed tomography (HRCT) is essential to improve diagnostic accuracy [4,5,6]. However, in many ILDs, clinical setting and HRCT appearances alone are insufficient to provide a definitive diagnosis [6,7]. Histopathological confirmation is essential for many ILDs, with specimen acquisition method including surgical lung biopsy (SLB) that traditionally was considered the gold standard. However, since the first application in lung periphery over a decade ago, the transbronchial lung cryobiopsy (TBLC) has been widely adopted for ILD diagnosis [8]. As the evidence base for this procedure continues to grow, some centers are moving away from conventional SLB, in favor of the less invasive TBLC.

Decision-making regarding the optimal approach to lung biopsy balances the benefits and risks of available techniques. SLB is the gold standard for diagnostic yield, ranging between 93.5 and 98.0% [9,10,11]. However, it is an invasive procedure requiring a multi-day hospital stay and placement of an intercostal catheter. The risk of exacerbation of ILD is high (6.1%) [12] and 30-day mortality is approximately 2% for elective procedures, which increases in high-risk groups. TBLC is less invasive than SLB and, while diagnostic yield is lower than SLB (approximately ~80%), risks of morbidity and mortality are lower [9,11].

A persistent challenge in TBLC practice in diagnosing ILDs has been unexplained heterogeneity reported for the key outcomes of diagnostic yield, bleeding and pneumothorax [9,11]. In addition, the emergence of newer cryoprobe sizes has introduced further variability, while data regarding their diagnostic performance and safety profiles remain limited and are currently derived largely from non-systematic and early observational studies. This narrative review synthesizes the current evidence regarding procedural adjuncts: radial probe endobronchial ultrasound (RP-EBUS), and cone-beam computed tomography (CBCT) for biopsy localization and technical factors that influence the diagnostic yield and safety of TBLC in ILD, with particular emphasis on the evolving role of smaller-diameter cryoprobes.

## 2. History and Evolution of Cryotherapy in Interventional Pulmonology

Cryotherapy has its origins in the mid-19th century, when Arnott first demonstrated that controlled freezing could induce tissue destruction [12]. Advances in refrigerants through the early 20th century—particularly the introduction of liquid nitrogen and nitrous oxide—enabled deeper and more reliable tissue freezing [13,14]. Modern cryoprobes emerged in the 1960s with the development of liquid nitrogen and Joule–Thomson–based systems, making internal applications feasible [15,16]. The first use of cryotherapy in the tracheobronchial tree was reported in the late 1960s, with subsequent experimental and early clinical studies in the 1970s establishing its safety and morphological effects on airway tissues [17,18,19].

Initial pulmonary applications relied on repeated freeze–thaw cycles that produced delayed necrosis and sloughing of devitalized tissue, making cryotherapy useful for tumor debulking and airway recanalization in patients with obstructing endobronchial disease [20,21]. Over time, improvements in probe design expanded its role within interventional pulmonology. Cryoadhesion enabled immediate cryorecanalization and cryoextraction of tumors, blood clots, mucus plugs, and foreign bodies [8,22,23].

The most significant evolution has been the development of TBLC, which emerged in the 2000s as a minimally invasive method for obtaining larger, better-preserved lung specimens than conventional forceps biopsy. TBLC is now widely used in the evaluation of ILD [24,25,26], marking the transition of cryotherapy from a destructive modality to a central diagnostic and therapeutic tool in modern interventional pulmonology.

## 3. Literature Search Strategy

In this narrative review, the databases searched were PubMed and Google Scholar. We included studies from January 2020 to January 2026. The search terms used were transbronchial lung cryobiopsy, interstitial lung disease, cryoprobes 1.7 mm and cryoprobes 1.1 mm, safety profile and diagnostic yield. Only two systematic reviews were found within the last five years that included cryoprobes sizes: 2.4 mm and 1.9 mm. Single-centered and comparative studies were found for cryoprobes of 1.7 mm and 1.1 mm. No systematic review was found for the diagnostic and safety profile for cryoprobes of 1.7 and 1.1 mm in ILD. The aim of this review was to keep our search mainly limited to ILD. We excluded studies that primarily involved other indications such as peripheral pulmonary lesions, lung transplant assessment, and mediastinal lesions.

## 4. The Current Role of Transbronchial Lung Cryobiopsy in ILD Diagnosis

TBLC in ILD has evolved into a validated, guideline-supported alternative to SLB for patients with undiagnosed ILD who require histopathology. Its role is anchored in diagnostic performance, tissue quality, safety, and impact on multidisciplinary discussion (MDD).

Over the past decade, multiple societies have issued consensus statements aimed at standardizing TBLC performance in ILD [7,27,28,29,30], and the European Respiratory Society (ERS) has recently convened a task force to develop evidence-based recommendations regarding its role in patients with undiagnosed ILD [31]. High-confidence final diagnoses can be achieved in most patients undergoing TBLC [29,31], and the addition of TBLC data to MDD significantly increases the proportion of ILD and idiopathic pulmonary fibrosis (IPF) diagnoses made with high diagnostic confidence [25,32]. In the study by Troy et al., a diagnostic pattern was identified in 90.8% of TBLC samples, with 70.8% histopathologic agreement with SLB using guideline-refined patterns and a weighted κ of 0.70 (95% CI 0.55–0.86) [29]. Similar findings have been reported in other indirect comparative studies, with an overall TBLC diagnostic yield of 82.8% [10].

According to ERS guidelines, TBLC may be considered in patients with undiagnosed ILD who are otherwise eligible for SLB, provided the procedure is performed in experienced centers (conditional recommendation, “very low” certainty of evidence) [31]. Although TBLC yields slightly lower diagnostic confidence than SLB, the task force emphasizes that its lower rate of serious adverse events, shorter hospitalization, and reduced cost outweigh this limitation in appropriately selected patients [31]. TBLC is particularly valuable in individuals with severe respiratory impairment, significant comorbidities, or rapidly progressive ILD, in whom SLB may pose unacceptable risk or potentially accelerate disease progression [31]. Expert consensus further suggests that TBLC may be used to obtain histologic data for MDD when local expertise is available, with the choice between TBLC and SLB guided by benefit–risk assessment and institutional experience [7]. In cases where TBLC is nondiagnostic, repeat TBLC or SLB may be considered. A summary of major society guidelines is provided in the following Table 1.

## 5. Technical Determinants of Diagnostic Yield and Safety

In recent years, the standardization of TBLC procedure in ILD was first evaluated and proposed by Hetzel and his group in 2018 [27], and subsequently multiple chest societies in the world aligned similar recommendations. The standardization of TBLC ensures safety which is essential to obtain sufficient tissue samples and avoid early procedure termination that would affect the number of biopsies taken during the procedure.

Prophylactic airway occlusion is recommended during TBLC to prevent blood contamination of adjacent airways. An endobronchial blocker should be positioned in the target lobe, preferably outside the endotracheal tube (ETT) to maintain ventilation and bronchoscope mobility. As previously described, the ETT is advanced over the bronchoscope and secured, after which the blocker snare is tightened around the bronchoscope tip to guide it into the target lobe for deployment [24,35]. The blocker should be tested for leaks under saline before the procedure begins.

After deployment, the ETT is advanced over a flexible bronchoscope with a large therapeutic channel. Patient tolerance may be assessed by briefly occluding the target lobe for 3–4 min; although not formally studied, this maneuver simulates post-biopsy obstruction and may identify patients at risk of desaturation. Real-time fluoroscopy should be used in all cases to guide cryoprobe placement.

**Figure 1:**
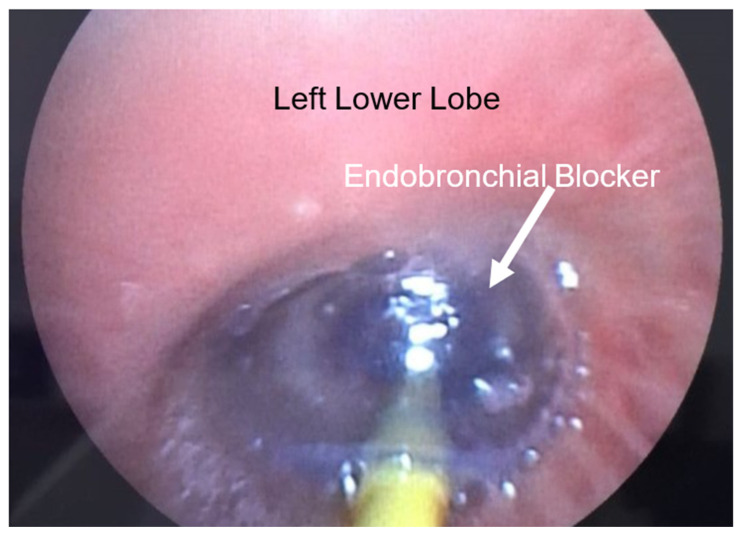
The endobronchial blockade (Arndt bronchial blocker, 7.0 French) with the balloon inflated in the left lower lobe, providing endoscopic confirmation that it is correctly positioned.

A carbon dioxide-cooled cryoprobe (1.7, 1.9, or 2.4 mm) is advanced under fluoroscopy until resistance is met, or the pleura is reached, then withdrawn approximately 1 cm to 2 cm and activated for 3 to 5 s. The cryoprobe and bronchoscope are removed en bloc. This technique samples parenchyma near the secondary lobule and subpleural region while minimizing pneumothorax and bleeding risk [27]. Immediately after removal, the bronchial blocker should be inflated to occlude the biopsied airway (Figure 1).

The specimen is thawed in room temperature saline to release the tissue, then transferred to formalin. The bronchoscope is reintroduced promptly to confirm blocker position and deflate it when appropriate. Hemostasis measures—iced saline, suction, tamponade, vasoconstrictors, blocker re-inflation, or patient repositioning—are applied as needed. Once hemostasis is secured, additional biopsies may be obtained. Most studies suggest that three biopsies, ideally from two different segments of the same lobe, are sufficient for diagnosis of diffuse ILD and different lobes for fibrotic ILD. This will be discussed in the next section.

### 5.1. Sampling Strategy for Fibrotic ILD vs. Diffuse ILD

The optimal sampling strategy for TBLC depends on the underlying ILD phenotype, particularly the degree of radiographic and histopathologic heterogeneity. The type of ILD diagnosis can be significantly influenced by both the heterogeneity of disease and the distribution of parenchymal pathology. Current TBLC guidelines recommend obtaining a minimum of three biopsies from two different sites, rather than a single location, with samples taken either from different segments of the same lobe or from different lobes [27]. This approach is especially important in fibrotic ILD, where pathological variability is more pronounced and differential diagnosis is more challenging. Ravaglia et al. reported discordant histologic patterns in nearly 30% of cases when comparing samples from different sites [26], with a corresponding improvement in diagnostic yield when biopsies were obtained from two sites rather than one. This variability reflects the well-recognized inter-lobar heterogeneity—where different lobes may show distinct histopathologic patterns—and intra-lobar heterogeneity, in which adjacent segments within the same lobe may harbor different patterns of fibrosis. It is the combination of inter-lobar, and intra-lobar heterogeneity seen in fibrotic ILD. The ability of TBLC to capture this heterogeneity is therefore essential for accurate diagnosis. However, sampling multiple lobes may increase the risk of pneumothorax and bleeding, particularly in fibrotic ILD where traction bronchiectasis and pleural distortion are common.

In contrast, in patients with diffuse radiographic abnormalities involving both upper and lower lobes, or in those with a clear apical–basal gradient, TBLC is more commonly performed in different segments within the same lobe. This strategy reduces procedural risk while still improving diagnostic yield, as biopsies from different segments of a single lobe have been shown to outperform sampling from the same segment alone [4]. Thus, tailoring the sampling approach to the radiographic pattern—multi-lobe sampling for heterogeneous fibrotic ILD versus multi-segment sampling within a single lobe for diffuse ILD—maximizes diagnostic accuracy while balancing procedural safety.

### 5.2. Impact of Probe Size and Freezing Time in Diagnostic Yield

The biophysical interaction between cryoprobe diameter, freezing kinetics, and resultant ice-ball geometry is a primary determinant of tissue volume and histologic adequacy in TBLC. Diagnostic performance is strongly linked to achieving a minimum specimen diameter of approximately 5 mm, which typically encompasses one or more secondary pulmonary lobules and provides sufficient alveolated parenchyma for pattern recognition in ILD [27]. Because ice-ball radius increases proportionally with both probe surface area and freezing duration, optimization of these parameters is essential to consistently generate specimens above this diagnostic threshold.

Historically, 1.9 mm and 2.4 mm reusable cryoprobes have been the most widely utilized before the introduction of new smaller probes. The larger probe sizes yield diagnostically adequate tissue; however, their freezing time requirements differ due to differences in thermal mass and conductive surface area. In a retrospective analysis, the 2.4 mm probe achieved optimal specimen size with 4–6 s of freezing, whereas the 1.9 mm probe required 6–8 s to generate comparable tissue volume [26]. This compensatory extension of freezing time with smaller probes effectively offsets their reduced diameter, enabling adequate ice-ball formation and preservation of alveolated architecture. Although the 2.4 mm probe consistently produces larger specimens, one study demonstrates a significantly greater mean surface area compared with the 1.9 mm probe (24.6 vs. 22.0 mm^2^, *p* < 0.001) and no significant differences were observed in pathological or multidisciplinary diagnostic yield [36]. These findings support the concept that once a minimum tissue volume is achieved, incremental increases in sample size confer limited additional diagnostic benefit.

The introduction of smaller single-use cryoprobes with outer diameters of 1.1 mm and 1.7 mm has further refined procedural flexibility. Comparative data show no statistically significant differences between the 1.9 mm and 1.7 mm probes in pathological diagnostic yield, MDD, diagnostic yield, adverse event rates (bleeding or pneumothorax), or sampling adequacy metrics—including alveolated tissue percentage, crush artifact burden, and biopsy dimensions [37]. These findings indicate that probe diameter alone is not the primary determinant of diagnostic performance; rather, diagnostic adequacy is governed by achieving a target specimen size through appropriate freezing time, precise probe positioning, and adherence to standardized sampling protocols.

## 6. Bleeding Prevention with Endobronchial Blocker or Endobronchial Balloon

Early adoption of TBLC technology (pre-2019) was associated with higher bleeding rates, which may in part reflect the lack of routine endobronchial blocker use. The Kheir 2022 [11] systematic review provides a representative baseline for bleeding risk in TBLC for ILD prior to the widespread adoption of endobronchial blockers and or endobronchial balloons. Bleeding was the most common complication, occurring in roughly 30% of procedures (95% CI 20–41%). Severe bleeding and procedure-related mortalities were uncommon. Because most included studies did not use endobronchial blockers, the review reflects outcomes from conventional TBLC techniques and provides a baseline against which newer airway-occlusion strategies can be compared. The review included studies that used heterogeneous procedural techniques, most of which did not employ pre-placed bronchial balloons or dedicated endobronchial blockers, relied on older 1.9–2.4 mm cryoprobes, and varied substantially in sedation methods and airway control strategies [11].

In 2018 ACCP guidelines made a recommendation to perform TBLC with a bronchial blocker either through an endotracheal tube or rigid bronchoscope (Ungraded Consensus-Based Statement) [27]. The evidence was provided by an observational study by Dhoorria et al. which reported the incidence of such bleeding was significantly lower in patients who underwent TBLC with prophylactic balloon placement (2/114 [1.8%]; *p* < 0.001) [38]. In this study a flexible cryoprobe (outer diameter 1.9 mm) was used with a freezing time of 3–6 s.

Another retrospective single-center study by Tavener et al. included one hundred and twenty-six patients who underwent TBLC. Significant bleeding was reported in 10 cases [7.9%]. These cases required balloon blocker reinflation for over 20 min, admission to ICU, packed red blood cell transfusion, bronchial artery embolization, resuscitation, or procedural abandonment. Significant bleeding was associated with traction bronchiectasis on HRCT (OR 7.1, CI 1.1–59.1, *p* = 0.042), a TBLC histological pattern of UIP (OR 4.0, CI 1.1–14, *p* = 0.046), and the presence of medium-large vessels on histology (OR 37.3, CI 6.5–212, *p* < 0.001). Of note, BMI ≥ 30 (*p* = 0.017) and traction bronchiectasis on HRCT (*p* = 0.025) were significant multivariate predictors of longer total bleeding time (*p* = 0.01). This study showed that other factors should be taken into consideration when assessing potential bleeding risk despite endobronchial blocker use. In this study biopsies were performed with a 2.4 mm flexible cryoprobe (Erbecryo II; ERBE, Tübingen, Germany) and taken 10 mm from the pleura under fluoroscopic guidance with a 4–7 second freezing time [39].

In 2024, Bian et al. [40], in a prospective, single-center, randomized controlled trial, patients with suspected ILD were enrolled and assigned to pre-placed balloon and non-pre-placed balloon. There were no significant differences in severe bleeding between the non-pre-placed balloon group and pre-placed balloon group (1.6% vs. 0.8%; adjusted *p* = 0.520); however, more moderate bleeding occurred in the non-pre-placed balloon group (26.4% vs. 6.4%, adjusted *p* = 0.001), as well as more use of hemostatic medication (28.0% vs. 6.4%, adjusted *p* = 0.001). More samples could be acquired in the pre-placed balloon group than in the non-pre-placed balloon group (3.8 ± 0.9 vs. 3.1 ± 0.9, *p* < 0.001) [40]. The study used a 7Fr endobronchial balloon occlusion catheter (Fogarty–type balloon), The endobronchial balloon (EBB) mainly shifts bleeding from moderate to mild/none, but does not clearly change severe bleeding events. However, the use of the endobronchial balloon did not affect the diagnostic yield in either group. The cryoprobe used was 1.9 mm cryoprobe with the freezing time of 5–6 s.

A retrospective study by Deasy et al. (2021) [41] included twenty five patients who underwent TBLC, of which twelve procedures used endobronchial balloon blockers (EBB). EBB subjects had significantly less moderate or severe airway bleeding (8.3% vs. 38.5%) despite higher biopsy rates in the EBB group, 2.9 (2–4) vs. 2.4 (1–4) in the non-EBB group. No severe airway bleeding occurred in the EBB group. No statistical difference in diagnostic yield was found after a multidisciplinary meeting (MDM) between the EBB group and the non-EBB group [41]. In this study, the investigators used a 1.9 mm cryoprobe with a freezing time of 4–6 s.

Overall, placing endobronchial blockers or endobronchial balloons did not affect the diagnostic yield but decreased bleeding severity when performing TBLC in ILD.

## 7. Imaging and Localization Adjuncts, Including Fluoroscopy, Radial Probe EBUS, and Cone-Beam CT

### 7.1. Probe-to-Pleura Distance by Using Fluoroscopy

**Figure 2:**
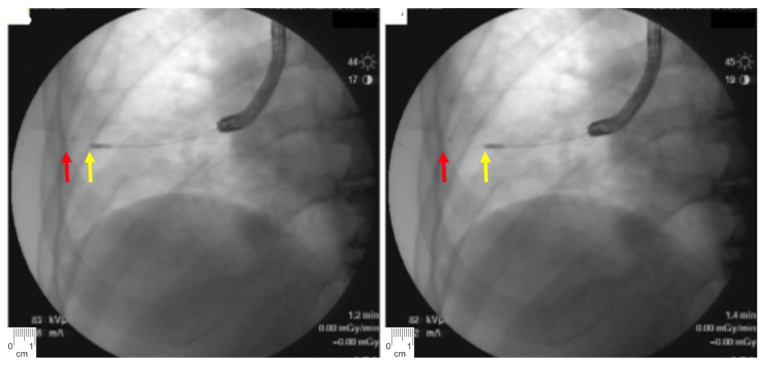
Using fluoroscopy to estimate the cryoprobe-to-pleura distance. Red arrows indicate pleural lining, and yellow arrows indicate the cryoprobe tip. The left image indicates cryoprobe-to-pleura distance when resistance is felt on insertion of the cryoprobe. The right image demonstrates retraction of the cryoprobe an additional centimeter prior to tissue sampling.

The distance between the activated cryoprobe and the pleura is a critical determinant of both diagnostic yield and procedural safety. This distance can be estimated in several ways. To date, all cases have been performed under fluoroscopic guidance. Lateral airways are preferred because the cryoprobe intersects the pleura perpendicularly, allowing the operator to use the approximately 1–2 cm cryoprobe tip is visible on fluoroscopy (Figure 2) to gauge the withdrawal distance before activation. In anteriorly or posteriorly directed airways, lightly pinching the catheter at the bronchoscope’s working channel while the tip contacts the pleura allows measurement of the withdrawal distance. Additionally, fluoroscopic visualization of the cryoprobe “catching” on the internal aspect of the ribs during respiration can confirm pleural contact in these orientations.

### 7.2. Use of Radial Probe Endobronchial Ultrasound (RP-EBUS) When Performing Transbronchial Lung Cryobiopsy

Evidence supporting the use of RP-EBUS during TBLC in ILD remains limited. One of the earliest evaluations was a small prospective study from a Polish group in 2018 that enrolled twenty patients and used RP-EBUS in place of fluoroscopy. The probe was advanced distally until the visceral pleura was occasionally visualized, then slowly withdrawn to identify a safe biopsy zone based on the distance from pleura and hilar vessels. Biopsies were performed at an estimated 1–2 cm from the pleural surface. The study reported a diagnostic yield of approximately 80% and a complication rate of 5%, suggesting that RP-EBUS may serve as a feasible alternative when fluoroscopy is unavailable [42].

In a multicenter prospective study by Inomata et al. [43], eighty-seven patients with diffuse parenchymal lung disease were evaluated, including forty-nine who underwent RP-EBUS. During RP-EBUS, the “blizzard sign” was identified as a whitish acoustic shadow of air-containing lung tissues, and the “dense sign” was identified as a darker and more homogeneous signal with irregularly distributed mottling and occasional linear hyperechoic areas. TBLC sites were selected as those in which RP-EBUS demonstrated blizzard or dense signs and an absence of blood vessels, with radiographic guidance.

Dense RP-EBUS signs corresponded to areas of consolidation on high-resolution CT, and specimens obtained from these regions demonstrated significantly higher pathological confidence compared with samples showing blizzard signs (*p* < 0.01) or samples from patients who did not undergo RP-EBUS (*p* < 0.05). RP-EBUS guidance was also associated with reduced bronchial bleeding and shorter procedure times (*p* < 0.01). Biopsy sites were selected based on the presence of blizzard or dense signs and the absence of adjacent vascular structures, integrating ultrasound and radiographic guidance to optimize sampling [43].

Additional support for RP-EBUS in TBLC comes from a large prospective cohort study by Oh et al. [44], which included 201 patients undergoing protocolized TBLC with a 2.4 mm cryoprobe. RP-EBUS was used to assess vascularity within the target airway and to identify the location of both the visceral and interlobar pleura. The thin, flexible ultrasound probe enabled visualization of pleural structures that may lie parallel to the airway, particularly when the airway is situated approximately 1 cm from the chest wall. Fluoroscopy was used in all cases for additional localization. The pneumothorax rate was 4.9%, and severe bleeding occurred in only one patient (0.5%), indicating that RP-EBUS may enhance procedural safety when incorporated into a multimodal guidance strategy [44].

Taken together, available studies suggest that RP-EBUS may improve the safety and diagnostic performance of TBLC by enabling identification of pleural boundaries, assessing airway vascularity, and guiding biopsy toward sonographically and radiographically representative regions. However, the overall evidence base remains sparse, with most studies limited by small sample sizes, heterogeneous methodologies, and frequent reliance on adjunctive fluoroscopy. Larger, standardized prospective studies are needed to clarify the specific contribution of RP-EBUS to TBLC outcomes and to determine its role relative to fluoroscopy and CBCT in ILD evaluation.

### 7.3. Use of Cone-Beam CT When Performing Transbronchial Lung Cryobiopsy

Cone-beam computed tomography (CBCT) has become an important adjunct to TBLC in the evaluation of ILD. By providing real-time three-dimensional imaging, CBCT improves visualization of the cryoprobe relative to the pleura, enhancing procedural control compared with traditional fluoroscopy. This improved spatial accuracy has been associated with reduced rates of pneumothorax and bleeding, while also increasing the likelihood of sampling disease-representative parenchyma in subpleural areas and improving diagnostic yield.

A key advantage of CBCT is its ability to safely guide biopsies closer to the visceral pleura, often with probe-to-pleura distances of less than 1 cm. This represents a departure from earlier fluoroscopy-based recommendations advising a 1–2 cm distance to minimize pleural injury. In ILD patterns with predominant subpleural involvement, such as usual interstitial pneumonia or limited-extent fibrotic disease, accurate sampling of the most pathologically representative regions can be challenging under fluoroscopy. CBCT overcomes this limitation by enabling precise localization of the cryoprobe tip and confirmation of its trajectory before activation. This capability is particularly valuable in cases where disease is patchy or confined to narrow subpleural zones, where small deviations in probe placement may significantly affect diagnostic yield.

Several studies have demonstrated the clinical benefits of CBCT-guided TBLC [45,46]. In a retrospective cohort of 120 patients, Ali and colleagues compared TBLC performed under fluoroscopy (TBLC-F) with CBCT-guided TBLC (TBLC-CBCT). The CBCT group achieved a mean probe-to-pleura distance of 5.1 ± 2.3 mm with an average of 4.0 ± 0.3 CBCT spins. Pneumothorax occurred more frequently in the fluoroscopy group (9.7%) than in the CBCT group (1.7%), and grade 2 bleeding was observed only in the fluoroscopy cohort. Diagnostic performance also favored CBCT, with final multidisciplinary diagnoses achieved in 95% of CBCT cases compared with 89% of fluoroscopy-guided procedures [45]. Similarly, Benn et al. reported a diagnostic yield exceeding 90%—comparable to SLB—using CBCT guidance, with no peri- or post-procedural complications and a mean probe-to-pleura distance of 4.2 ± 1.3 mm [46].

Traditional fluoroscopy requires maintaining a 1–2 cm probe-to-pleura distance to compensate for limited depth perception and reduce pleural injury risk. In contrast, CBCT provides real-time 3-D visualization, allowing safe placement within <1 cm of the pleura. This precision enables sampling of the most pathologically representative subpleural regions—particularly important in UIP and limited-extent fibrotic ILD—while simultaneously reducing pneumothorax and bleeding. Thus, whereas fluoroscopy-guided TBLC generally favors a greater pleural distance to mitigate pleural injury risk, CBCT-guided TBLC permits more precise subpleural targeting and may improve sampling of diagnostically relevant fibrotic lung tissue [44].

CBCT has also shown utility in patients with interstitial lung abnormalities (ILA) or ILD affecting less than 15% of the lung parenchyma, where precise targeting is essential. Ravaglia and colleagues [10] demonstrated a pathological diagnostic yield greater than 90% in this population, with nearly 80% of diagnostic samples obtained on the first biopsy pass. Although pneumothorax occurred in 27.8% of cases—reflecting the inherently subpleural nature of the disease—no severe adverse events were reported. Despite these promising results, the widespread adoption of CBCT-guided TBLC remains limited by the scarcity of CBCT-equipped bronchoscopy suites and concerns regarding radiation exposure. These constraints highlight the need for further technological refinement and broader access to advanced imaging platforms to fully realize the benefits of CBCT in ILD diagnostics. In addition, the availability of CBCT platforms and disposable ultrathin cryoprobes may remain limited in low- and middle-income settings, where procedural cost and access to advanced bronchoscopy infrastructure may influence implementation of these approaches.

## 8. The Evolution of Cryoprobe Size

Early in the adoption of TBLC, particularly in the late 2010s, pneumothorax rates were reported as high as 26%, with severe hemorrhage occurring in up to 18% of cases [47,48,49]. During this period, the 2.4 mm and 1.9 mm cryoprobes were commonly used. A meta-analysis suggested a trend toward higher pneumothorax rates with the 2.4 mm probe compared with the 1.9 mm probe [50]. Several series using the 2.4 mm probe-reported pneumothorax rates of 20–28% potentially related to biopsies taken within 1 cm of the pleura [10,51]. Most likely these complications were related to the lack of standardization of TBLC procedure in ILD. These complications seemed to have reduced after the TBLC procedure standardization—focusing on the use endobronchial blocker or endobronchial balloon, the use of fluoroscopy and established airway (ETT or under rigid bronchoscopy)—led to reduced risk in both pneumothorax and bleeding. In a recent systematic review, the reported complication rates included bleeding in 12% of cases and pneumothorax in 5% but there was substantial heterogeneity in outcomes, largely attributable to modifiable procedural factors [52]. This review suggested that pneumothorax risk increased with use of the 2.4 mm probe, multiple-lobe sampling, reduced mean DLCO, and general anesthesia, while moderate-to-severe bleeding was more frequent with the larger probe and higher bleeding scores.

Rodrigues and colleagues [9] compared safety of TBLC to SLB—for the TBLC, the pooled complication rates included 9.9% for significant bleeding (reduced to 6.9% in high-volume centers), 5.6% for pneumothorax requiring chest tube drainage. Despite the TBLC procedure standardization, the risk for complications is still present.

The diagnostic yield for TBLC in ILD when using the cryoprobes 2.4 mm and 1.9 mm has been well described. Early comparative evidence between TBLC and SLB emerged in 2016, when Ravaglia et al. [10] conducted a large single-center retrospective analysis in an ILD cohort. This study included 150 video-assisted thoracoscopic surgery (VATS) biopsies and 297 TBLC samples from separate patients. A histopathological diagnosis was obtained in approximately 99% of patients who underwent VATS and 83% of those who underwent TBLC. However, beyond the inherent limitations of retrospective analysis, this study did not incorporate MDD to establish a final integrated clinical–radiological–pathological diagnosis.

The 2019 COLDICE study by Troy et al. [29,53] represents a pivotal milestone in establishing the diagnostic performance of TBLC in ILD. In this rigorously designed paired-sample investigation, TBLC demonstrated high diagnostic utility, achieving concordance rates of approximately 70% with SLB for histopathologic patterns and 76% for final multidisciplinary diagnoses, thereby confirming its capacity to provide clinically actionable tissue. Usual interstitial pneumonia (UIP) was the predominant histopathologic pattern in the cohort, identified in roughly half of all participants (33/65, 50.8%), underscoring the study’s emphasis on evaluating TBLC’s ability to capture UIP-defining subpleural features. The study demonstrated a diagnostic agreement between TBLC and SLB in patients with ILD, with both procedures performed during the same anesthetic episode. The authors suggested that a stepwise approach—using TBLC as the initial procedure and reserving SLB for inconclusive TBLC cases—could reduce patient burden while achieving a diagnostic yield comparable to immediate SLB, although they emphasized the need for long-term data and larger prospective cohorts to confirm equivalence of these strategies.

In 2024, the COLD study [54] became the first randomized trial to directly compare two diagnostic strategies for ILD: a step-up approach (TBLC first, followed by SLB only if TBLC was inconclusive) versus immediate SLB. The TBLC-first strategy significantly reduced chest tube drainage, length of hospital stay, pain, and serious adverse events, while maintaining a similar overall diagnostic yield. In this trial, the diagnostic yield was 82% with TBLC alone, 88% with immediate SLB, and 89% with the step-up strategy. Supplementary data detailing the specific cryoprobe sizes used were not accessible, limiting procedural granularity.

In a 2024 systematic review, Lachowicz et al. [52] evaluated the diagnostic yield of TBLC in ILD including studies up to April 2022, the mean number of samples obtained per patient was 3.4 ± 0.85, yielding an overall diagnostic rate of 81%. The review identified substantial heterogeneity in outcomes, largely attributable to modifiable procedural factors. Only two cryoprobe sizes—2.4 mm and 1.9 mm—were represented, and diagnostic performance was influenced by probe diameter, anesthesia modality, baseline respiratory function, and the presence of a multidisciplinary ILD assessment prior to biopsy.

In another systematic review, Rodrigues and colleagues [9] compared the diagnostic performance and safety of TBLC and video-assisted thoracoscopic surgery (VATS) across 43 studies. Of these, 23 studies evaluated TBLC diagnostic yield following MDD, demonstrating a pooled diagnostic yield of 76.8%, which increased to 80.7% in high-volume centers performing ≥ 70 TBLC procedures. Ten studies assessed VATS, with a pooled diagnostic yield of 93.5%. The most reported freezing time ranged from 3 to 6 s [9]. Collectively, these findings reinforce that while VATS maintains the highest diagnostic yield, TBLC offers a favorable balance of diagnostic performance.

In the mid-2020s, the introduction of single-use, smaller-diameter cryoprobes marked an evolution in TBLC. These newer probes are thought to enhance maneuverability and improve tactile feedback when the pleura is contacted, potentially reducing the risk of pneumothorax and bleeding; however, supporting evidence remains limited and derives primarily from small observational studies. With the 1.7 mm cryoprobe, the procedural workflow remains largely unchanged from that used with larger probes, and tissue retrieval continues to require en-bloc extraction. More recently, the smallest 1.1 mm cryoprobe paired with an oversheath system has been developed. This platform allows specimen retrieval through the bronchoscope’s working channel—similar to forceps biopsy—while the oversheath protects the channel and permits repeated probe passage without removing the bronchoscope. This design enables continuous airway visualization and immediate management of bleeding, with the oversheath remaining wedged to provide tamponade. Despite these advantages, the single-use nature of the probe and the added oversheath introduce higher procedural costs and a learning curve that may influence adoption across centers with varying experience and resource availability.

The available data evaluating smaller cryoprobes (1.7 mm and 1.1 mm) remain limited, and no comprehensive systematic reviews have specifically addressed their diagnostic performance in ILD. It remains unclear whether procedural modifications—such as increasing freezing time—are required when using these smaller probes to obtain tissue samples of sufficient size and quality for accurate diagnosis of ILD (Table 2—Summary of studies with cryoprobes 1.7 mm and 1.1 mm).

## 9. The Evaluation of TBLC in ILD with 1.7 mm Cryoprobe

### 9.1. Diagnostic Yield

Across small published studies, the 1.7 mm cryoprobe has demonstrated diagnostic performance comparable to the conventional 1.9 mm probe. In a prospective randomized trial of sixty patients, Ravaglia et al. [37] found no statistically significant difference in pathological diagnostic yield between the two probe sizes (100% for the 1.9 mm probe vs. 93.3% for the 1.7 mm probe; *p* = 0.718). A 2022 single-center analysis of seventy-seven TBLC procedures similarly showed that the 1.7 mm single-use probe produced diagnostically adequate specimens when biopsies were obtained at an optimal 0.5–1.0 cm distance from the pleura, with tissue quality equivalent to that obtained using the larger reusable probe [59]. A 2025 Japanese cohort employing a standardized 5 s freeze protocol reported a 96% specimen adequacy rate, with cryobiopsy findings substantially increasing multidisciplinary diagnostic confidence—particularly in idiopathic pulmonary fibrosis (83.3%) and hypersensitivity pneumonitis (87.5%) [55]. Fujiwara et al. [58] likewise reported an overall diagnostic yield of 82% in fifty patients, with no age-related differences in tissue adequacy.

### 9.2. Safety Profile

Studies showed that the 1.7 mm cryoprobe maintains a comparable complication profile, comparable to that of the 1.9 mm probe. In the randomized trial by Ravaglia et al. [37], rates of pneumothorax and mild-to-moderate bleeding were similar between probe sizes. The 2022 a single-center analysis likewise reported no excess risk of pneumothorax or bleeding when using the 1.7 mm probe, even when sampling closer to the pleura [59], but this should be interpreted with caution given all the required chest tubes. In the 2025 Japanese cohort, pneumothorax occurred in 4% of cases and moderate bleeding in 12%, despite the use of a standardized 5 s freeze time [55]. Fujiwara et al. [58] also observed low overall complication rates, with no severe bleeding. Taken together, pneumothorax occurred more frequently in the 1.7 mm cryoprobe group, although differences were not statistically significant. This trend may reflect deeper subsegmental access and reduced probe-to-pleura distance.

### 9.3. Technical and Procedural Characteristics

Beyond diagnostic yield and safety, these small cohorts suggested procedural advantages associated with the 1.7 mm cryoprobe. Its smaller diameter improves maneuverability within narrower or more angulated bronchial segments, potentially expanding access to subsegmental targets in patients with complex airway anatomy. The 2022 single-center study demonstrated that when biopsies were obtained at an optimal 0.5–1.0 cm distance from the pleura, the 1.7 mm probe produced specimens of comparable size and histologic adequacy to those obtained with the 1.9 mm probe [59]. The 2025 Japanese cohort showed that even with a shorter 5 s freeze time, the probe yielded diagnostically adequate tissue in nearly all cases [55]. Fujiwara et al. [58] further demonstrated consistent performance across age groups, suggesting broad applicability in routine ILD evaluation. Overall, the 1.7 mm cryoprobe offers technical versatility without compromising tissue quality.

Despite these encouraging findings, the current evidence base remains limited by small sample sizes, single-center designs, heterogeneous procedural protocols, and the absence of systematic reviews or large multicenter trials; therefore, these results should be interpreted with caution and validated in broader, more rigorous studies.

## 10. The Evaluation of TBLC in ILD with 1.1 mm Cryoprobe

### 10.1. Diagnostic Yield

The randomized controlled trial by Bian et al. [56], which enrolled 224 patients with suspected ILD, found statistically equivalent diagnostic yields between the 1.1 mm and 1.9 mm probes (80.4% vs. 79.5%). Although the smaller probe generated specimens with reduced surface area, tissue weight and histologic quality remained comparable, confirming that diagnostic adequacy is preserved. Complementing these findings, Zhang et al. [57] reported a diagnostic yield of 88.5% in a real-world cohort of 52 ILD patients, with MDD identifying a broad distribution of ILD subtypes, including hypersensitivity pneumonitis, idiopathic interstitial pneumonias, and rare ILDs. Collectively, these two studies suggest that the 1.1 mm cryoprobe provides a diagnostic yield; however, the evidence remains very limited to establish definitive conclusions regarding diagnostic yield.

### 10.2. Safety Profile

The 1.1 mm cryoprobe offers an acceptable and often favorable complication profile. In FROSTBITE-1 [60] (Safety of a Sheath Cryoprobe Bronchoscopic Transbronchial Biopsy Technique), which included 50 patients undergoing bronchoscopy for diffuse parenchymal lung disease, lung-transplant allograft assessment, or focal lesions, no severe bleeding events or major complications were observed, even during early adoption when the first ten cases were performed under rigid bronchoscopy. In the randomized trial by Bian et al. [56], the 1.1 mm probe was associated with lower rates of moderate bleeding compared with the 1.9 mm probe (6.2% vs. 17.0%), while pneumothorax rates were similar, with a nonsignificant trend toward higher incidence in the smaller-probe group. Finite-element modeling in the same study suggested that the 1.1 mm probe produces greater tension and stress on the target lobe, offering a biomechanical explanation for subtle differences in complication patterns. Real-world data from Zhang et al. [57] further support the probe’s safety, with severe bleeding in only 3.8% of cases and pneumothorax in 1.9%; none required chest tube placement. Together, these findings indicate that the 1.1 mm cryoprobe is safe and well-tolerated, with bleeding risk potentially lower than that of larger probes.

### 10.3. Technical and Procedural Characteristics

**Figure 3:**
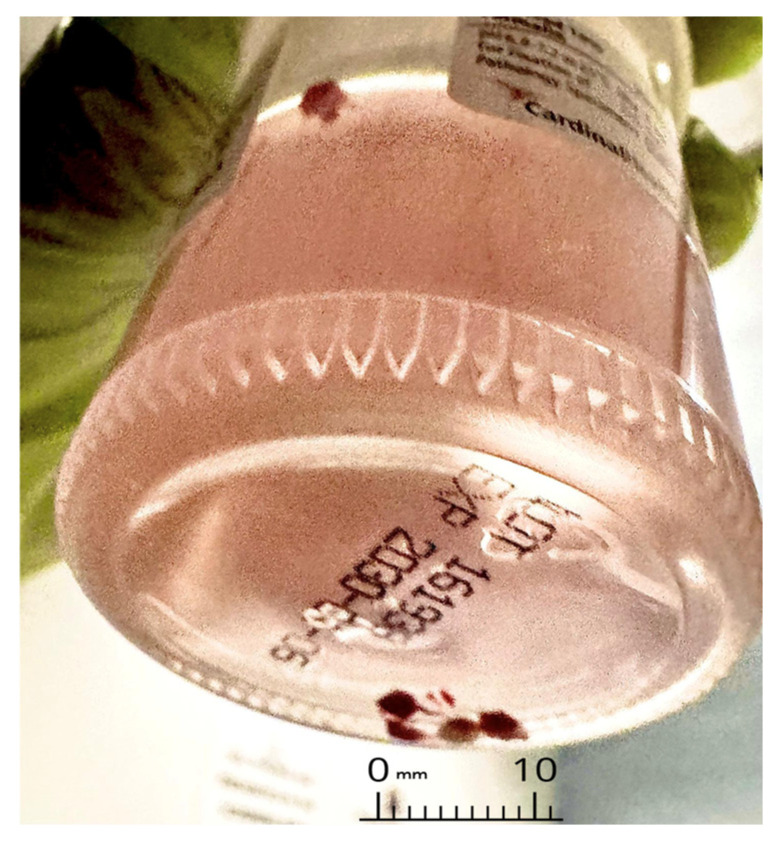
Transbronchial lung cryobiopsies in formalin sampled with the 1.1 mm cryoprobe with oversheath and freezing time of 4 s.

The 1.1 mm cryoprobe introduces several important technical innovations that distinguish it from traditional cryobiopsy platforms. Its defining feature is the oversheath system, which allows biopsy specimens to be retrieved through the bronchoscope’s working channel, eliminating the need for en-bloc removal of the scope and thereby preserving continuous airway visualization [60]. This design enables immediate identification and management of bleeding, while the oversheath remains wedged to provide tamponade and protects the working channel during repeated probe passage. In FROSTBITE-1, freezing time (FT) began at 4 s, with incremental increases of 1 s up to a maximum of 8 s if tissue was inadequate, and three biopsies were obtained for ILD evaluation [60] and increasing FT did not increase bleeding. In the Bian et al. trial [56], although the 1.1 mm probe produced smaller surface-area specimens, tissue weight and histologic quality were preserved, and finite-element modeling revealed unique biomechanical properties that may influence complication patterns. Zhang et al. [57] reported freezing times of 3–9 s, with an average of 3.5 ± 1.2 specimens per procedure and a median specimen diameter of 5 mm, confirming that clinically meaningful tissue can be obtained despite the smaller probe diameter. These technical characteristics—particularly through-scope retrieval, continuous visualization, and flexible freeze-time protocols—highlight the 1.1 mm cryoprobe as a versatile and workflow-enhancing tool, though its single-use design and learning curve may influence adoption across centers. (Figure 3—TBLC with cryoprobe 1.1 mm-showing a specimen of ~5 mm).

## 11. Evidence Gaps and Future Directions

Despite the promising procedural advantages of the 1.1 mm and 1.7 mm cryoprobes—including improved maneuverability, through-scope retrieval, and enhanced airway visualization—significant evidence gaps remain. Current data are derived primarily from small, single-center studies with heterogeneous protocols, and although early results suggest that smaller probes may achieve diagnostic yields approaching those of conventional 1.9 mm probe, the evidence is not yet sufficient to confirm diagnostic equivalence. Larger, multicenter comparative studies are needed to determine whether these ultrathin probes can reliably match the diagnostic performance of standard TBLC tools across diverse ILD populations. Additionally, future research should clarify how probe size interacts with factors such as biopsy location, probe-to-pleura distance, imaging guidance, and operator experience—variables that may influence both diagnostic yield and complication patterns. Ultimately, while smaller cryoprobes align with broader trends toward minimally invasive standardized TBLC practice, their routine incorporation into ILD diagnostic algorithms will require more robust evidence, ideally through randomized trials and systematic reviews.

## 12. Discussion

The role of TBLC in the diagnostic evaluation of ILD has expanded substantially over the past decade, supported by evidence demonstrating meaningful diagnostic concordance with SLB when interpreted within MDD. Landmark paired-sample studies such as COLDICE have shown that TBLC can provide histopathologic patterns and final diagnoses that align closely with SLB, reinforcing its utility as a less invasive alternative in appropriately selected patients [29]. Building on this foundation, contemporary research has shifted toward optimizing procedural safety while preserving diagnostic performance, particularly through the development and adoption of smaller-diameter cryoprobes. However, the true clinical impact of these innovations remains uncertain, as the evidence base is limited by the absence of randomized controlled trials and systematic reviews. directly comparing probe sizes within ILD diagnostic pathways.

TBLC performed with conventional larger probes (2.4 mm and 1.9 mm) consistently achieves diagnostic yields exceeding 80%, with an acceptable safety profile in experienced centers [37,57]. The introduction of small; cryoprobes 1.7 mm and the 1.1 mm cryoprobe—featuring an oversheath that enables through-scope specimen retrieval, continuous airway visualization, and potentially improved bleeding control—represents a meaningful procedural advancement [60]. Early feasibility data and one randomized trial suggest that diagnostic yield with the 1.1 mm probe is comparable to that of the 1.9 mm probe, and that histologic adequacy is preserved despite smaller surface-area specimens [56]. Nevertheless, the available studies remain relatively small and heterogeneous, and they are not yet sufficient to establish equivalence to conventional cryoprobes or to SLB within ILD diagnostic algorithms.

The impact of probe size on pneumothorax risk remains an area of ongoing uncertainty. Pneumothorax occurred more in the 1.7 mm cryoprobe group, although differences were not statistically significant. This trend may reflect deeper subsegmental access and reduced probe-to-pleura distance inherent to smaller-diameter probes. The pneumothorax rates for the 1.1 mm probe are broadly comparable to those observed with larger probes [56]. Pneumothorax risk is influenced not only by probe size but also by biopsy location, probe-to-pleura distance, sampling strategy, fluoroscopic or CBCT guidance, operator experience, and disease distribution [9,52,59]. These procedural and anatomical variables likely play a more substantial role in complication patterns than probe size alone.

As TBLC continues to evolve, the introduction of smaller-caliber probes—including the 1.7 mm and 1.1 mm platforms—offers the potential to refine safety and expand procedural versatility. Yet, given the limited number of high-quality comparative studies, the absence of systematic reviews, and the heterogeneity of existing data, these innovations should be interpreted cautiously. Large, multicenter randomized trials—such as the ongoing FROSTBITE-2 study—are expected to provide critical evidence regarding the diagnostic performance, safety, and optimal clinical role of ultrathin cryoprobes in ILD evaluation [61]. Until such data are available, TBLC probe selection should remain individualized, balancing diagnostic needs, patient-specific risk factors, and operator expertise.

Finally, adjunctive imaging tools such as RP-EBUS and CBCT have been incorporated into TBLC workflows to enhance biopsy localization and potentially improve safety. RP-EBUS can help confirm parenchymal positioning and avoid inadvertent sampling of large vessels, while CBCT provides real-time three-dimensional guidance that improves the accuracy of probe-to-pleura distance assessment—an important determinant of pneumothorax risk [9,52,59]. Although these modalities appear to support more precise biopsy targeting and may reduce complications in selected settings, their use remains highly variable across institutions. Differences in equipment availability, procedural expertise, and workflow integration mean that the adoption of RP-EBUS or CBCT should be individualized by each center, balancing potential benefits with resource constraints and operator experience. Current evidence suggests that these tools can aid diagnosis and enhance procedural control, but their routine use in TBLC has not yet been validated through randomized trials or systematic reviews, and therefore they should be considered as supportive rather than essential components of ILD diagnostic pathways.

## 13. Conclusions

The smaller 1.1 mm and 1.7 mm cryoprobes represent a promising advancement in TBLC within the interventional pulmonology field, but further multicenter comparative studies are needed before its routine role in ILD diagnostic algorithms can be firmly established. The adoption of RP-EBUS or CBCT should be individualized by each center, balancing potential benefits with resource constraints and operator experience.

## Figures and Tables

**Table 1:** Major society guideline recommendations regarding the role of transbronchial lung cryobiopsy (TBLC) in the diagnostic evaluation of interstitial lung disease.

Guideline Document	Year	Recommendation Regarding TBLC	Recommendation Strength/Certainty of Evidence	Key ILD-Specific Considerations
ATS/ERS/JRS/ALAT Clinical Practice Guideline: *Idiopathic Pulmonary Fibrosis (an Update) and Progressive Pulmonary Fibrosis in Adults* [33]	2022	Suggests that TBLC may be regarded as an acceptable alternative to SLB for histopathologic diagnosis in patients with ILD of undetermined type when performed in experienced centers.	Conditional recommendation; very low-quality evidence.	Recommends integration of TBLC findings within MDD, procedural standardization, and careful patient selection. Notes that relative contraindications include severe physiologic impairment, significant pulmonary hypertension, marked hypoxemia, and elevated bleeding risk.
ERS Guideline: *European Respiratory Society Guidelines on Transbronchial Lung Cryobiopsy in the Diagnosis of Interstitial Lung Diseases* [31]	2022	Suggests TBLC as a replacement test in selected patients with undiagnosed ILD who are considered eligible for SLB, and as an option in selected patients not considered eligible for SLB. Suggests step-up SLB following non-informative TBLC when additional histopathologic data remain clinically indicated.	Conditional recommendations; evidence generally of very low certainty.	Emphasizes operator training, institutional experience, multidisciplinary interpretation, quality assurance, and individualized assessment balancing diagnostic yield against procedural risk. Recognizes lower mortality, shorter hospitalization, and lower procedural invasiveness relative to SLB, despite somewhat lower diagnostic yield.
CHEST Guideline and Expert Panel Report: *Transbronchial Cryobiopsy for the Diagnosis of Interstitial Lung Diseases* [7]	2020	Supports TBLC as a less invasive diagnostic alternative to SLB in appropriately selected patients with ILD requiring histopathologic evaluation. Evidence suggests that TBLC contributes meaningfully to MDD-based diagnosis, although histopathologic diagnostic yield remains somewhat lower than SLB.	Two graded recommendations and four consensus-based statements; overall certainty of evidence limited.	Recommends standardized procedural protocols including fluoroscopic guidance, airway control strategies, prophylactic bronchial blockade, and performance in centers with appropriate procedural expertise to optimize safety and diagnostic yield.
ATS/ERS/JRS/ALAT Clinical Practice Guideline: *Diagnosis of Idiopathic Pulmonary Fibrosis* [34]	2018	No recommendation was made for or against TBLC because of insufficient evidence and lack of procedural standardization.	No consensus recommendation.	Identified substantial heterogeneity in diagnostic yield, complication rates, specimen quality, and procedural techniques across centers, emphasizing the need for prospective studies and procedural standardization.

Abbreviations: ALAT, Latin American Thoracic Association; ATS, American Thoracic Society; ERS, European Respiratory Society; ILD, interstitial lung disease; JRS, Japanese Respiratory Society; MDD, multidisciplinary discussion; SLB, surgical lung biopsy; TBLC, transbronchial lung cryobiopsy.

**Table 2:** Summary of studies with cryoprobes 1.7 mm and 1.1 mm.

Study	Study Design	Population and Indication	Sample Size	Cryoprobe Size and Type	Freezing Time	Number of Biopsies	Diagnostic Yield or Diagnostic Confidence	Moderate or Severe Bleeding	Pneumo-thorax	Chest Tube Requirement	Main Limitations
Ravaglia et al. (Pulmonology, 2023) [37]	Prospective randomized trial	ILD-only cohort; suspected diffuse parenchymal lung disease	*n* = 60	1.7 mm single-use vs. 1.9 mm reusable cryoprobe	5–7 s standardized protocol	NR	Pathological diagnostic yield 93.3–100%; no significant difference between probe sizes	Mild-to-moderate bleeding rates comparable between groups; no severe bleeding reported	Comparable between groups	NR	Single-center study; limited sample size; freezing protocol effects not independently analyzed
Koike et al. (J Thorac Dis, 2025) [55]	Retrospective single-center analysis	ILD-only cohort; suspected interstitial lung disease	*n* = 25	1.7 mm single-use cryoprobe	Standardized 5–6 s protocol	NR	Specimen adequacy 96%; improved MDD diagnostic confidence	Moderate bleeding 12%; no severe bleeding	4%	NR	Small retrospective cohort; no comparator arm
Bian et al. (Lung, 2024; randomized controlled trial) [56]	Prospective randomized controlled trial	ILD-only cohort; suspected ILD	*n* = 224	1.1 mm vs. 1.9 mm cryoprobes	3–5 s (1.1 mm) vs. 5–7 s (1.9 mm)	NR	Equivalent diagnostic yield between probes (80.4% vs. 79.5%)	Moderate bleeding lower with 1.1 mm probe (6.2% vs. 17.0%)	Similar between groups; nonsignificant trend toward higher pneumothorax with 1.1 mm probe	NR	Long-term outcomes unavailable; specimen size differed between groups
Zhang et al. (Frontiers in Medicine, 2026) [57]	Retrospective observational study	ILD-only cohort	*n* = 52	1.1 mm cryoprobe	3–9 s	Mean 3.5 ± 1.2 biopsies	Diagnostic yield 88.5% with adequate alveolated tissue acquisition	Severe bleeding 3.8%	1.9%	No chest tube placement required	Single-center retrospective design
Fujiwara et al. (Okayama Medical Center, Japan; J Thorac Dis, 2024–2025) [58]	Retrospective single-center study	ILD-only cohort	NR	1.7 mm single-use cryoprobe	Fixed 5–6 s institutional protocol	NR	High pathological adequacy with preserved alveolated architecture and strong diagnostic confidence after MDD	No severe bleeding reported	Low frequency reported	NR	Single-center experience; limited generalizability

## Data Availability

No new data were created or analyzed in this study. Data sharing is not applicable to this article.
